# Attractiveness of employment sectors for physical therapists in Ontario, Canada (1999-2007): implication for the long term care sector

**DOI:** 10.1186/1472-6963-12-133

**Published:** 2012-05-29

**Authors:** Michel D Landry, Robyn Hastie, Känecy Oñate, Brenda Gamble, Raisa B Deber, Molly C Verrier

**Affiliations:** 1 Doctor of Physical Therapy Division, Duke University Medical Centre, Box 104002, Durham, North Carolina, USA; 2 Department of Physical Therapy in the Faculty of Medicine, University of Toronto, Toronto, ON, Canada; 3 Department of Health Policy and Management in the Gillings School of Global Public Health, University of North Carolina at Chapel Hill, Chapel Hill, North Carolina, USA; 4 Department of Health Policy, Management and Evaluation (HPME) in the Faculty of Medicine, University of Toronto, Toronto, ON, Canada; 5 Faculty of Health Sciences, University of Ontario Institute of Technology, Oshawa, ON, Canada

**Keywords:** Physical therapy, Health human resources, Workforce

## Abstract

**Background:**

Recruiting and retaining health professions remains a high priority for health system planners. Different employment sectors may vary in their appeal to providers. We used the concepts of inflow and stickiness to assess the relative attractiveness of sectors for physical therapists (PTs) in Ontario, Canada. Inflow was defined as the percentage of PTs working in a sector who were not there the previous year. Stickiness was defined as the transition probability that a physical therapist will remain in a given employment sector year-to-year.

**Methods:**

A longitudinal dataset of registered PTs in Ontario (1999-2007) was created, and primary employment sector was categorized as ‘hospital’, ‘community’, ‘long term care’ (LTC) or ‘other.’ Inflow and stickiness values were then calculated for each sector, and trends were analyzed.

**Results:**

There were 5003 PTs in 1999, which grew to 6064 by 2007, representing a 21.2% absolute growth. Inflow grew across all sectors, but the LTC sector had the highest inflow of 32.0%. PTs practicing in hospitals had the highest stickiness, with 87.4% of those who worked in this sector remaining year-to-year. The community and other employment sectors had stickiness values of 78.2% and 86.8% respectively, while the LTC sector had the lowest stickiness of 73.4%.

**Conclusion:**

Among all employment sectors, LTC had highest inflow but lowest stickiness. Given expected increases in demand for services, understanding provider transitional probabilities and employment preferences may provide a useful policy and planning tool in developing a sustainable health human resource base across all employment sectors.

## Background

The delivery of health care services relies on an appropriate and sustainable health human resource base. Although medical technological advances are occurring with tremendous momentum, there continues to be a necessity to have a health workforce to provide services when and where they are most required. Health professionals practice and contribute across a care continuum, and a priority of health system policy and decision makers is to ensure stability among the workforce so that health system can function effectively to meet objectives: as such, workforce recruitment and retention is an essential component of an effective health system. This may be particularly important as the global workforce is aging [1-3] and as health care demand appears to be rising across the gradient of high, middle and low income countries [4-7].

Similar to many other countries, health human resource (HHR) policy and planning is a priority for health systems managers and planners across Canada [8-13]. The demand for health services has been reported to be rising across the care continuum [14-18]. However, a stable trend in Canada has been the ongoing shift in delivering care away from the hospital and towards the home and community sectors [19,20]. The public policy arguments used to justify this shift often include cost savings, appropriateness of providing community-based services closer to where individuals and their families live, and the evolution of services that can be feasibly provided in the community [21].

The shift toward the community not only affects *where* care is delivered, it also affects *how* care is provided and by *whom*. For instance, different employment settings may mean different wages, working conditions, and differences (subtle and “not-so-subtle”) in the structure of providing care, including altered physical work environment, reporting structures, and emphasis on interprofessional models of care [22,23]. It may be reasonably straightforward for a family physician who operates a hospital-based ambulatory clinic to physically transfer her practice into the community; but on the other hand, it may be complicated, or socially undesirable, for a nurse who has worked for years in intensive care to transfer his employment to community based care due to complex organizational and funding processes, job interruption and re-training requirements. Variations in the attractiveness of employment settings may ultimately affect the ability of employment sectors to recruit and retain providers [24].

One way to assess the appeal or attractiveness within an employment sector is to assess overall attrition or turnover rates of providers. It might be assumed that low staff turnover implies an attractive employment setting, and high turnover indicates an unattractive one, although there may be individual factors that apply for each health provider group. In a study of the nursing workforce in Ontario (Canada), Alameddine et al. developed the concepts of ‘stickiness’ and ‘inflow’ as useful proxy measures for relative attractiveness within the spectrum of employment sectors [24]. They defined stickiness as the transitional probability that a provider will remain in a sector year-to-year, and inflow as the proportion of new providers in a sector. Using relative attractiveness as a measure, the authors suggested that despite overall shrinkage in the nursing workforce in all hospital types, the hospital sector remained highly attractive among nurses as assessed by high stickiness. Moreover, the authors found that although the community sector expanded, it was a relatively unattractive sector for nurses as evidenced by low stickiness. The authors concluded that there was considerable variation in relative attractiveness across all employment sectors for nurses, and that the concept of stickiness could be applied for other health human workforces for which linked longitudinal data are available.

In this study, we focus on physical therapists (PTs). We examined physical therapy employment sectors based on four primary logics: first, the physical therapy workforce in Canada has not been well documented in the literature; second, the demand for physical therapy and rehabilitation services has been reported to be increasing in the province [15,17,23,25,26]; third, “PTs” are among the largest provider group within rehabilitation [27-29]; and fourth, funding streams and delivery models in which physical therapists practice have experienced much change over the last decade [30,31]. There were almost 17,000 PTs in Canada in 2010. According to the Canadian Institutes for Health Information [32], approximately 78% of PTs in the country were female, the average age was 41.7, and approximately 70% practice in either ‘general practice’ or “musculoskeletal and integumentary systems.’ The education system for PTs has changed in the last few decades from a college diploma, to a bachelor of physical therapy degree, to now a Master’s entry-level to practice degree in all 14 educational institutions. Eligibility to practice is not based on educational degree, but rather on being registered to practice within one of the 13 jurisdictions. In order to be eligible to practice as a physical therapy, one must have graduated from a accredited institution or its equivalent and have passed a national competency examination. The province of Ontario has the largest overall population, and the largest number of PTs in the country (5,600 PTs, or about a third, of all PTs in the country in 2010). Landry et al. [33] reported that between 1991 and 2005, there was a 11.6% growth in the health human resource ratios across Canada, as measured by the number of PTs per 10,000 population. However, in contrast, the health human resource ratio in the United States grew by 61.3% between 1995 and 2005. In their comparative analysis, Landry et al. noted that although the growth among PTs in the United States was greater than in Canada, this should not be interpreted as one country outperforming the other because evidence-based workforce benchmarks do not exist in physical therapy. Recently Zimbelman et al. [34] reported that, “demand for physical therapy will outpace the supply of PTs with the United States. Shortages are expected to increase for all 50 states through 2030.” (page 1021). Although a similar study has not yet been performed in Canada, it is likely to be similar to the US projections given similarities in context.

The purpose of this study was to use the concepts of inflow and stickiness developed by Alameddine et al. [24], to assess relative attractiveness of employment sectors for physical therapists in Ontario with a view to understand more fully the extent to which different employment sectors were able to retain PTs between 1999 and 2007.

## Methods

As noted above, we defined stickiness as the transitional probability that a physical therapist working in a given sector in year “*t*” will remain in the same sector in year “*t + 1*” [24]. In this study, employment sector refers to one of four sectors self-reported by physical therapists to be their primary employer. These four sectors are: ‘hospital’, ‘community’, ‘long term care’ (LTC), or ‘other’. Each sector contained a variety of sub-sectors. For example, physical therapists classified as working in the hospital sector may be employed in a variety of hospital settings (e.g., rehabilitation, acute care, etc.). Given this operational definition of an employment sector, the term stickiness should not be considered to be the opposite of turnover because a physical therapist moving from one employer to another within the same sector would represent turnover for that employer but that physical therapist would stay, or still be “sticky,” within that sector. The number of physical therapists working in sector *y* in year *t* is denoted as N_*y,t*_ , the number of physical therapists working in sector *y* in year (*t* + 1) is expressed as N_*y,t*+1_, and the number of physical therapists working in sector *y* in both years *t* and _*t*+1_ is denoted as N_*y,t&t*+1_. The formula for stickiness is thus expressed as follows:

(1) $$Stickiness_{t}{}_{tot+1}=\left[\left(N_{y,t\&t+1}/N_{y,t}\right)\right]\times 100\%$$

Similarly, the concept of ‘inflow’ was defined as the percentage of physical therapists that are working in a particular sector in year “*t*”, but who were not working in that sector in year “*t*-1”.

(2) $$Inflow_{t}=\left[\left(N_{y,t}-N_{y,t\&t}{}_{-1}\right)/N_{y,t}\right]\times 100\%$$

For instance, if 1000 physical therapists worked in hospitals in 2010, of whom 800 had worked there in the year 2009, then the inflow_2010_ would be:

(3) $$\left(\frac{1000-800}{1000}\right)\times 100\%=20\%$$

In this study, we analyzed aggregated stickiness and inflow, defined as average values of each variable across the 8-year period from 1999 to 2007, as a way to explore the extent to which providers tended to stay in a particular employment sector over time. High stickiness can represent an attractive sector, and alternatively, low stickiness can denote low attractiveness. If a sector had high inflow and high stickiness, we interpreted this as an attractive and expanding setting because of an increased volume of new providers working in the setting, and because the providers already in that sector tended to remain. Alternatively, if a sector had low inflow and low stickiness, we interpreted this as an unattractive and shrinking sector because not only were there few new providers entering the sector, the ones who were working in that sector did not remain over time. High inflow and low stickiness, could represent a fairly unattractive setting (due to low stickiness), but a growing sector (due to the high inflow). Low inflow and high stickiness, on the other hand, could represent a highly attractive sector (due to high stickiness) but one that is not necessarily growing (due to low inflow). Based on these operational definitions, the terms inflow and stickiness can only be interpreted as relative variables, which must be weighed against overall changes in the size of particular employment sectors. Inflow will be higher in sectors that are expanding for a variety of reasons (e.g., policy changes, funding infusion), and can result from new additions to the workforce (e.g., new graduates, internationally educated providers), and/or from physical therapists returning to the workforce as well as those switching between sectors.

A longitudinal dataset was created with data from the College of Physiotherapists of Ontario (CPO). Annual registration with the CPO is a prerequisite to practice as a physical therapist in the province of Ontario. Upon first registration with the CPO, each individual physical therapist is given a unique registration number, and this unique identifier remains with them for their career. Similar to other regulated health professionals, physical therapists have an incentive to keep their registration active even if they are temporarily out of work, or on leave, to avoid the requirements involved with reinstating a lapsed registration. The annual registration process requires payment, along with self-reporting of information regarding location of employment and hours of practice in the previous year. The variable of primary sector of employment/practice over the years was critical to determine inflow and stickiness in this study. While we recognize that some PTs may have more than one sector of employment, this longitudinal data set used ‘primary’ sector of employment as a key feature. Primary sector of employment is defined as the singular sector in which a registered physical therapist allocated the majority of their time. While we acknowledge that focusing only on primary employer may create a limitation in our analysis, it was a necessary step in examining translational probabilities.

Access to a de-identified registrant database was provided directly from the CPO. A sub-set of the data containing a specific set of variables for all physical therapists registered was created for each year from 1999 to 2007. The CPO database contains 42 employment sectors. These were collapsed into 4 predominant sectors that we defined as ‘hospital’, ‘community’, ‘long -term care (LTC)’ and ‘other’ and are similar to the categories used by Alamedine et al [24].

The ‘hospital’ sector was a collection of sub-sectors that were related to hospitals, or hospital-based care. ‘Community’ sector was defined as publicly-funded community based practices, or community organizations. ‘Long Term Care’ was defined as settings that were institutionally based, and where care was provided to persons who were admitted as residents of the facility. Finally, ‘other’ sector was a heterogeneous collection of sub-sectors, including private practices, consulting agencies, charities, and others. When an employment category did not appear to fit logically within any of the four categories, the research team reviewed and made a decision as to which category was best suited. This was a rare occurrence, and in all cases that it did occur, the ‘other’ category was used.

Registrants were assigned according to their self-identified primary place of employment. In 2002, the CPO altered the categories for primary place of work, and although many employment categories were similar, others differed and reflected the evolving role of physical therapists in the province. In order to address this methodological issue, we re-categorized the primary employment sector during the registration years 2002-2007 according to the pre-determined categories (1999-2002). Table 1 outlines the list of primary employment sectors used in the analysis.

**Table 1:** Definition of sectors of physical therapy employment

**Sector**		**Disaggregated Employment Categories**
‘Hospital’	1999-2003	Acute Care Hospital, Addiction Facility, Ambulatory Care Center, Facility for Mentally Challenged, General Hospital, Hospital Outpatient Program, Oncology Hospital, Pediatric Hospital/Facility, Physiotherapist Owned, Psychiatric Facility, Rehabilitation Hosp/Facility
	2004-2007	Complex Continuing Care, General Hospital Teaching or Mental Health Facility, Pediatric Hospital Facility, Rehabilitation Facility, Rehabilitation Hospital
‘Community’	1999-2003	Community Care Access Centre, Community Centre, Community Health Centre, Home Care Program, Physician Owned Clinic, Schedule 5/OHIP Restricted, Schedule 5/OHIP
	2004-2007	Community Care Access Centre, Community Health Centre, Home Visiting Agency, OHIP Clinic
‘Long Term Care’	1999-2003	Long Term Care Hosp/Facility, Nursing Home, Facility for the Aged, Retirement Home, Retirement Residence, Seniors Home
	2004-2007	Long Term Care Facility
‘Other’	1999-2003	Hydrotherapy-non physiotherapist owned, Consulting Firm/Agency, Fitness Centre, Government/Other Official Agency, Industry, Insurance, Other, Performing Arts Clinic, Pharmaceutical Company, Private Practice/Clinic, Private Rehab Agency, Professional/Health Association, Rehabilitation Consulting Firm, Retailer, School Board, Sports Association, The Arthritis Society, University/Educational Institution.
	2004-2007	Arthritis Society, Consulting Firm Agency, Government Other Official Agency, Industry, Other, Private Practice Clinic Owned, Private Practice Clinic, Professional Health Association, Retailer, School Board, University Educational Institution

The dataset for each year was merged to create a longitudinal dataset of all physical therapists registered from 1999 to 2007. However, due to data quality issues, the transitions between registration year 2002/2003 and 2003/2004 were omitted from this analysis. While we acknowledge that this presents a weakness within the study, we determined through trends analysis that the omission of these data did not alter in any meaningful way the overall findings of the study.

Data analyses were performed using SAS software (SAS Institute Inc, Cary, NC, USA). A series of frequency distributions were first performed in order to determine the distribution of physical therapists across sub-sectors of employment for each year. Then 1-year transitional probabilities for each employment sector over the 1999-2007 period were created, along with aggregated mean values by sector.

Ethics approval was provided by the Ethics Review Board at the University of Toronto.

## Results

### Size and inflow trends by employment sector (1999 to 2007)

There were 5003 physical therapists registered to practice in Ontario in 1999, and 6064 in 2007, indicating an increase of 21.2%. Table 2 outlines the trends in the size of each employment sector in Ontario between 1999 and 2007, and the extent to which the sector expanded or contracted. The trends indicate that all sectors expanded during this time period, ranging from an increase of 11.6% in the “hospital” sector, to an increase of 76.0% in the “LTC” sector. The unadjusted growth percentage in the LTC sector must be viewed with the understanding that the LTC sector started from a relatively low base in 1999.

**Table 2:** Number of physical therapists by sector (1999-2007)

	**Hospital**	**Community**	**LTC**	**Other**	**Total**
Start (1999)	2267	764	129	1843	5003
End (2007)	2531	845	227	2461	6064
Change in Number	264	81	98	618	1061
(Start-End)					
Percent Change	11.6	10.6	76.0	33.5	21.2
(Start-End)					
Employment Sector Status	Expansion	Expansion	Expansion	Expansion	Expansion
(Expansion vs. Contraction)					

In Table 3, the annual ‘inflow’ results are presented across all employment sectors. Each sector has experienced an increased influx of new providers, but not all with the same proportional growth. Inflow represents the number of new workers, which is influenced by both the overall sector growth, and the need to replace those who left the sector. The percentage of inflow ranged from as low as 13.8% in the hospital sector, to as high as 32.0% in the LTC sector. The average annual inflow across all sectors during this study period was 20.7%. Using the inflow average as a cut-off point, the LTC was the only sector where expansion was above provincial average. The three other sectors would be considered below average, with the community sector being just below average expansion.

**Table 3:** Percentage inflow by year by sector (1999-2007)

	**Hospital**	**Community**	**LTC**	**Other**	**Total**
1999/00	12.8	21.6	23.1	21.8	19.8
2000/01	13.5	16.6	14.4	16.7	15.3
2001/02	14.2	16.8	20.5	11.9	15.8
2004/05	19.5	24.1	67.4	21.9	33.2
2005/06	13.4	22.6	40.5	17.2	23.4
2006/07	9.7	15.5	26.0	15.5	16.5
Mean	13.8	19.5	32.0	17.5	20.7

Note: The transition results for the 2002/2003 and 2003/2004 years were removed from the analysis due to poor data quality; LTC = Long Term Care.

### Stickiness trends by employment sector (1999 to 2007)

Table 4 outlines stickiness results across all sectors. The average annual stickiness value across all sectors during this study period was 81.4%, meaning that the majority of physical therapists across all sectors tended to stay in the same sector. This is a conservative estimate of stickiness, because the departures include both physical therapists that left the profession completely due to death or retirements (permanently existed the physical therapy workforce) and those who switched to another sector.

**Table 4:** Percentage stickiness by year by sector (1999-2007)

	**Hospital**	**Community**	**LTC**	**Other**	**Total**
1999/00	83.8	77.1	79.8	87.4	82.0
2000/01	86.0	77.5	75.2	90.2	82.3
2001/02	90.1	89.8	89.0	88.9	89.5
2004/05	85.5	66.2	50.4	82.6	71.2
2005/06	87.7	73.9	65.8	84.2	77.9
2006/07	91.0	84.8	80.0	87.1	85.7
Mean	87.4	78.2	73.4	86.8	81.4

Note: The transition results for the 2002/2003 and 2003/2004 years were removed from the analysis due to poor data quality; LTC = Long Term Care.

The hospital sector had the highest stickiness (or the ability to retain physical therapists year-to-year). Over the study period, an aggregated 87.4% of physical therapists employed stayed in that same sector, although they may have changed their place of employment from one hospital to another during this period. The ‘other’ sector had a comparatively high stickiness value of 86.8%. The full list of employment categories included in the ‘other’ category is included in Table 1, however it is notable that a majority (but not all) of PTs included in the ‘other’ category were employed in private-funded settings. The community and the LTC sectors had the lowest stickiness values, ranging from 78.2% to 73.4% respectively.

## Discussion

Two important findings emerged from this study. First, during the 8-year study period of 1999 to 2007, the absolute number of physical therapists working in Ontario across all employment sectors grew by 21.2%. We assess this proportional growth among physical therapists to be relatively low compared to growth in other non-physician and nursing workforces. For instance, according to the Canadian Institute for Health Information, between 2001 and 2008, respiratory therapists grew by 43%, speech language pathologists by 36%, social workers grew by 69%, and chiropractors by 33% [35]. These findings are consistent with other reports that have suggested that the overall supply of physical therapists has increased over time, but not necessarily as fast as other provider groups or the provincial population [27,31,35]. Expansion that occurred in the physical therapy workforce was in contrast to reports for nursing over a similar period. Alameddine et al. found that the hospital sectors contracted by 13.0%, and that the LTC, community and other sectors expanded by 10.8%, 9.4% and 10.8% respectively over a similar period of time [24]. Further research into the expansion of employment sectors, and the distribution between full-time, part-time and casual employees, among other regulated and unregulated health providers would provide useful comparative data for our results.

A second important finding is that we have identified differences across the care continuum regarding relative attractiveness of practice sectors for physical therapists. Our analysis has shown that the hospital sector had relatively low ‘inflow’ of new providers, but the highest stickiness (87.4%), suggesting that the hospital sector remains a highly attractive compared to other sector of employment for physical therapists. Viewed from the alternative perspective, just over 12% of physical therapists employed in hospitals in 1999 left this sector by 2007. However to our knowledge there are no known established evidence-informed benchmarks to clarify whether these data constitute high or low retention rates. Even with numerous provincial hospital closures and/or restructuring, along with rationing of human resources in the hospital sector over this study period, physical therapy human resources demonstrate, in our opinion, a fairly high degree of stability.

The hospital sector findings are in contrast to what we assess to be lower employment attractiveness in the LTC sector as evidenced by its high inflow and lower stickiness. This sector might be presumed to be unattractive because many providers entered or were already working in that the sector in 1999, but then left by 2007, indicating that this sector may be unable to retain providers relative to other sectors. Based on our data, although many new providers enter the LTC sector, close to 27% of the aggregated workforce in LTC left over time. Subsequent to our initial analysis, we have conducted a review of the profile of those who identify LTC as their primary sector of employment. We have founds anecdotally that an increasing proportion of PTs who were trained in low-income countries are practicing in LTC, and by 2007 approximately 25% of PTs educated in low-income countries, but who were registered with the CPO, practice in the LTC sector.

The published literature suggests that there are many factors that can influence a health provider’s decision to remain in a specific employment sector. Tett and Myers [36] suggested that job satisfaction was a strong predictor of intention to leave an employment setting [36]. Since the publication of their seminal work, many international researchers have explored workforce predictors, and have evaluated the extent to which job satisfaction can influence attrition rates across providers [37-41]. Collectively, these studies have outlined that there are a series of intrinsic and extrinsic factors that can influence or motivate rehabilitation providers to remain in a specific work setting. Specific to the retention of rehabilitation providers, Tran et al*.*[29], from an analysis of a series of expert panels, identified that there are three essential areas of critical development to ensure stability of a rehabilitation workforce including 1) quality of worklife and work environment, 2) financial incentive and marketing, and 3) professional development. There is little research to examine the specific factors that apply to rehabilitation providers in the LTC sector. However, McGilton et al*.* have suggested that an absence of clear roles for nurses within the LTC sector in Ontario “may undermine efforts to recruit and retain charge nurses, thereby, negatively affecting the quality of care” [42].

LeRoy et al. [43] describe many approaches that could also be used to improve the foundation in the long-term care sector, including the need to change the culture towards a more patient-centered approach. They argue that improving the culture of the sector would improve the working conditions among health profesisonals. Moreover, McGilton et al. [44] reported previously that job satisfaction among nurses in LTC were statistically related to supervisor support. It would seem logical therefore to assume that many of the issues that surround the LTC workplace may be related to the environment in which nurses, and all other health professional, work. Given that there is an increased reliance on the LTC sector to reduce admission and so-called re-admissions to hospitals, it might be useful for policy and decision makers to heed the mounting evidence [45-50] and improve on the sector’s weaknesses to ensure a stable and ‘sticky’ workforce.

It remains unknown if these stickiness values are within acceptable parameters for rehabilitation professionals. Neither is it possible, at this time, to judge whether the stickiness data represent low or high attrition and, possibly even more germane to HHR policy, whether the current situation of stickiness creates cause for concern in terms of providing appropriate and timely services. The absence of HHR indicators through which to interpret these findings ultimately limit the answers to these and other important workforce questions. However, our data do create scope for further investigations to develop sensitive indicators to inform the evolving field of HHR policy and practice.

We recognize some study limitations that constrain the extent to which the data can be interpreted. The longitudinal dataset categorized physical therapists according to their self-declared primary practice sector, and as such, for a physical therapist who practiced in a different hospital in each year of practice, our data would consider this individual to have remained in the hospital sector across the study period. Thus individual hospitals might have high turnover, but the aggregated provincial hospital sector would report very low turnover. That said, investigating the metrics of the hospital sector (i.e., percentage of part time and casual employees) constitutes an essential area where further research is needed to determine the stability of this large constituency of the physical therapy workforce. Another inherent limitations to this data analysis approach include the lack of consistency of multiple employment sectors. For instance, a physical therapist might have declared that his/her primary practice sector is hospital, but may have also been working for other employers making the attribution of behavior to the primary employer somewhat inflated. The study timeline of 1999 to 2007 represented a period of rapid growth and evolution in physical therapy in the province of Ontario, but given that another 5-year period has transpired since the analysis, it is critically important to further examine the extent to which the results and trends that we report remain accurate. The longitudinal dataset was specific to the PTs primary sector of employment. It is possible that PTs had multiple employers, however a strategic decision was made to focus on the setting in which a physical therapist allocated the majority of their time. A final limitation that is noteworthy is that the personal, political or even social factors may have influenced the decision among PTs to shift sectors. For instance, a change in martial or family status, or overall system changes including (but not limited to) hospital downsizing may have influenced decisions among practitioner more than the practice setting setting itself.

## Conclusions

During the 8-year period of 1999 to 2007, all physical therapy employment sectors expanded, albeit at different rates. The hospital sector appeared to be the sector with the lowest growth (lowest inflow), but remained a relatively attractive employment sector due to the ability to retain a fairly consistent physical therapy workforce year-to-year. On the other hand, despite highest overall proportional growth or inflow, more physical therapists in LTC migrate out of the sector compared to any other sector. This finding may be important for provincial policy because the LTC sector is growing at a much faster rate than others. Using our operational definitions of relative attractiveness, the ‘hospital’ and ‘other’ sectors appear to be the most attractive, followed by the community sector. The LTC sector is the least attractive employment sector across the care continuum in physical therapy. Given that the provincial health care system has been implementing reform that is shifting the locus of care from hospitals to community, these data signal that physical therapy HHR in the LTC sector may not be fully consistent with this paradigm shift. Our retrospective data do not clarify the reasons for differences in relative attractiveness across sectors; however, based on these data we signal the need to understand more fully the recruitment and retention factors. Exploring the factors that surround the LTC sector should be a priority, given the provincial government’s focus on this sector. Moreover, human resource policies for service outside hospitals should be developed with a view to improving the retention of physical therapists and other providers, and to ensure that LTC sector employs the appropriate human resource compliment to deliver appropriate and timely services. Given the expected increase in demand for rehabilitation service, further research is needed to assess the implication of these HHR findings for the ability of health systems to meet future demands, especially given the provincial health reform strategy which includes shifts from hospital to home and community.

## Abbreviations

CPO: College of Physiotherapists of Ontario; HHR: Health human resource; LTC: Long term care; PTs: Physical therapists.

## Competing interests

The authors declare that they have no competing interests.

## Authors’ contributions

MDL designed the study, participated in the data collection, analyzed the data, and wrote successive drafts of the manuscript. RH, KO and BG conducted the majority of the data analysis, and reviewed successive drafts of the manuscript. RD and MCV both participated in the design of the study, and reviewed and revised successive drafts of the manuscript. All authors read and approved the final manuscript.

## Pre-publication history

The pre-publication history for this paper can be accessed here:

http://www.biomedcentral.com/1472-6963/12/133/prepub
